# CADM1 inhibits squamous cell carcinoma progression by reducing STAT3 activity

**DOI:** 10.1038/srep24006

**Published:** 2016-04-01

**Authors:** Sabari Vallath, Elizabeth K. Sage, Krishna K. Kolluri, Sofia N. Lourenco, Vitor S. Teixeira, Suneeta Chimalapati, P. Jeremy George, Sam M. Janes, Adam Giangreco

**Affiliations:** 1 Lungs for Living Research Centre, University College London, UK

## Abstract

Although squamous cell carcinomas (SqCCs) of the lungs, head and neck, oesophagus, and cervix account for up to 30% of cancer deaths, the mechanisms that regulate disease progression remain incompletely understood. Here, we use gene transduction and human tumor xenograft assays to establish that the tumour suppressor Cell adhesion molecule 1 (CADM1) inhibits SqCC proliferation and invasion, processes fundamental to disease progression. We determine that the extracellular domain of CADM1 mediates these effects by forming a complex with HER2 and integrin α6β4 at the cell surface that disrupts downstream STAT3 activity. We subsequently show that treating CADM1 null tumours with the JAK/STAT inhibitor ruxolitinib mimics CADM1 gene restoration in preventing SqCC growth and metastases. Overall, this study identifies a novel mechanism by which CADM1 prevents SqCC progression and suggests that screening tumours for loss of CADM1 expression will help identify those patients most likely to benefit from JAK/STAT targeted chemotherapies.

Invasive, metastatic squamous cell carcinomas (SqCCs) of the lungs, head and neck, oesophagus, and cervix are responsible for over 400,000 deaths per year^1^. SqCC progression involves acquisition of epithelial-to-mesenchymal transition (EMT) phenotypes including enhanced tumour cell motility, increased cell proliferation, elevated cell invasiveness, and reduced cell adhesiveness, all of which contribute to uncontrolled tumour growth and multi-organ metastases^2^. These changes are accompanied by increased MMP production, reduced E-cadherin protein abundance, and elevated Twist1 and Snail expression^3^,^4^,^5^. Although they occur in distinct organs, the characteristics of human SqCCs are conserved and frequently involve mutations in TP53, PTEN, LKB1 and SOX2^6^. Despite this, a comprehensive understanding of the mechanisms that drive human SqCC progression remains elusive.

Cell adhesion molecule 1 (CADM1, also known as Necl2, TSLC1, IGSF4, RA175, and SynCam), an Immunoglobulin superfamily (Igsf) adhesion molecule, is a well-known tumour suppressor for a variety of cancers of epithelial origin^7^. CADM1 downregulation through epigenetic silencing or loss of heterozygosity accompanies increased tumour cell invasion and metastatic potential, making it an attractive candidate for regulating SqCC progression^8^,^9^,^10^. In lung adenocarcinoma CADM1 inhibits tumour cell proliferation via cytoplasmic band 4.1 and MAGuK protein interactions^11^. In contrast, the tumour suppressive effects of CADM1 in breast adenocarcinoma are dependent upon T cell–mediated immune surveillance^12^,^13^. In immortalized kidney cells, the extracellular domain of CADM1 binds the receptor tyrosine kinase HER3, reducing cell proliferation^14^. Finally, in colon adenocarcinoma CADM1 regulates hemidesmosome stability by increasing integrin α6β4 interactions, thereby reducing tumour cell motility^15^. Thus, the mechanisms by which CADM1 regulates disease progression are highly dependent on the tumour and tissue type in which it is expressed.

In this study we use patient samples, cell lines, and human tumour xenograft models to define a key functional role for CADM1 in SqCC progression. We demonstrate that the extracellular domain of CADM1 restricts tumour growth and metastases by interacting with HER2 and integrin α6β4 at the cell surface. We establish that this CADM1-HER2-Itgα6β4 signaling complex reduces downstream STAT3 activity, an important regulator of SqCC proliferation and invasion. We also show that disrupting STAT3 signaling with the JAK1/2 inhibitor ruxolitinib replicates these effects specifically in CADM1-null disease. These results suggest that screening SqCC tumours for loss of CADM1 expression will help identify patients at greatest risk of disease progression and most likely to benefit from JAK/STAT targeted chemotherapies.

## Materials and Methods

### Human tissue samples

Human tissue samples were obtained via video chip flexible auto fluorescence bronchoscopy with full informed consent from all subjects in accordance with UK and University College Hospital research ethical guidelines (REC Approval No. 06/Q0505/12). All sample collection was approved by University College Hospital Research ethics committee. Briefly, samples were identified using fluorescence imaging, biopsied, fixed, and processed for pathological diagnosis as previously described^16^. For immunostaining, samples were formalin fixed, paraffin embedded (FFPE), and sectioned at 5 μm. This study was carried out in accordance with the Declaration of Helsinki (2000) of the World Medical Association.

### Cell culture and viral transduction

A431 cells were transduced with a constitutively active luciferase reporter and a doxycycline-inducible CADM1-FLAG-IRES-GFP lentivirus. FLAG-tagged CADM1 constructs including full length, extracellular (∆EC), and cytoplasmic (∆CP) domain deletion isoforms were provided by Prof. Y Takai and generated as previously described^14^,^15^. CADM1 constructs were then cloned into a doxycycline-inducible lentivirus as previously described^14^,^17^. Luciferase lentivirus was obtained from Addgene for *in vivo* tracking studies. Lentivirus production, transduction, and cellular selection were performed according to standard conditions^17^. The A431 SqCC cell lines were obtained from ATCC and validated using STR profiling (LGC standards). Cells were maintained in DMEM (Sigma) plus 10% FBS and glutamine. Recombinant EGF was provided to cells used in immunoblot studies.

### Human Tumorigenesis Studies

*In vivo* tumorigenesis studies were performed in accordance with UK Home Office guidelines under license 70/7607. All experiments were approved by University College London Biological Safety and Biological resource ethics committees. Luciferase expressing SqCC cells (10^5^/animal) were injected into the tail vein of NSG mice and tumour engraftment assessed by measuring luciferase activity. Mice were randomized such that tumour burden was balanced for each group. Doxycycline (1 mg/ml) was administered to the drinking water from day 0. Administration of ruxolitinib (2.5 mg/animal in 100 ul water) was performed daily beginning on day five after administration of human tumour cells; drug was administered via oral gavage. Quantification of tumour burden and metastatic spread was assessed by monitoring luciferase activity via IVIS imaging on a weekly basis following 200 μl intraperitoneal injections of 2 mg/ml D-luciferin (Regis). Animals were injected with 10 mg/kg BrdU 1 hour prior to sacrifice.

### Migration assay

Single SqCC cell suspensions (5 × 10^4^ cells in αMEM/0.1%BSA) were pre-treated with drug or antibody for 1 hour at 4 °C, seeded onto 8 μm Transwell inserts (Corning), and incubated for 16 hours at 37 °C in media containing drugs or antibodies as indicated. Inserts were formalin fixed, stained with 1% crystal violet, and imaged to assess cell migration. Stained, migrated cells were subsequently dissolved in 10% glacial acetic acid with migrated cell abundance quantified by UV-VIS spectroscopy.

### Proliferation assay

Cells (5 × 10^4^ in DMEM/10%FBS) were grown as indicated and XTT metabolic activity (corresponding to total cell abundance) was determined as per the manufacturer’s instructions (Promega, UK).

### Biochemical Methods

Cells were lysed in RIPA (Sigma) or immunoprecipitation lysis buffer (Thermo fisher) for immunoblotting and immunoprecipitation. Antibodies used were directed against CADM1 (chicken, MBL), FLAG (M2, Sigma Aldrich), ITGA6 (rabbit, R&D, Abcam), ITGB4 (Abcam), HER2 (Rabbit, Abcam), HSC70 (Santa Cruz), phospho-STAT3, total STAT3 (Cell Signalling), and tubulin (Cell signaling). For immunoprecipitation experiments, cell lysates (500 μg) were blocked, incubated with antibodies directed against FLAG (to pull down CADM1), HER2, or integrin α6, mixed with Protein A beads, washed and immunoprecipitated according to described methods^14^,^15^.

### Immunostaining

Paraffin embedded sections of human and murine tissues were subjected to haematoxylin and eosin (H&E) or immunofluorescence staining with antibodies directed against keratin 5 (rabbit, Abcam), CADM1 (chicken, MBL), E-cadherin (HECD1, mouse, Abcam), BrdU (rat, Abcam), Luciferase (rabbit, Abcam), keratin 14 (rabbit, Covance), and phospho-STAT3 (Cell Signalling). Detection of apoptotic cells was via TUNEL staining (Promega). Immunostained cells were fixed using 4% paraformaldehyde and incubated with antibodies to CADM1 (chicken, MBL), ITGA6 (rabbit, R&D), and HER2 (Rabbit, Cell Signalling).

### Statistical analyses

Statistical analysis was performed using GraphPad Prism Software (La Jolla, CA). A student’s t test was applied for comparisons between two groups (unpaired, two tailed). Two-way ANOVA with Bonferroni post-test analyses were used to compare differences in tumour growth over time between each group.

## Results

### CADM1 loss is associated with increased SqCC progression

**Figure 1:**
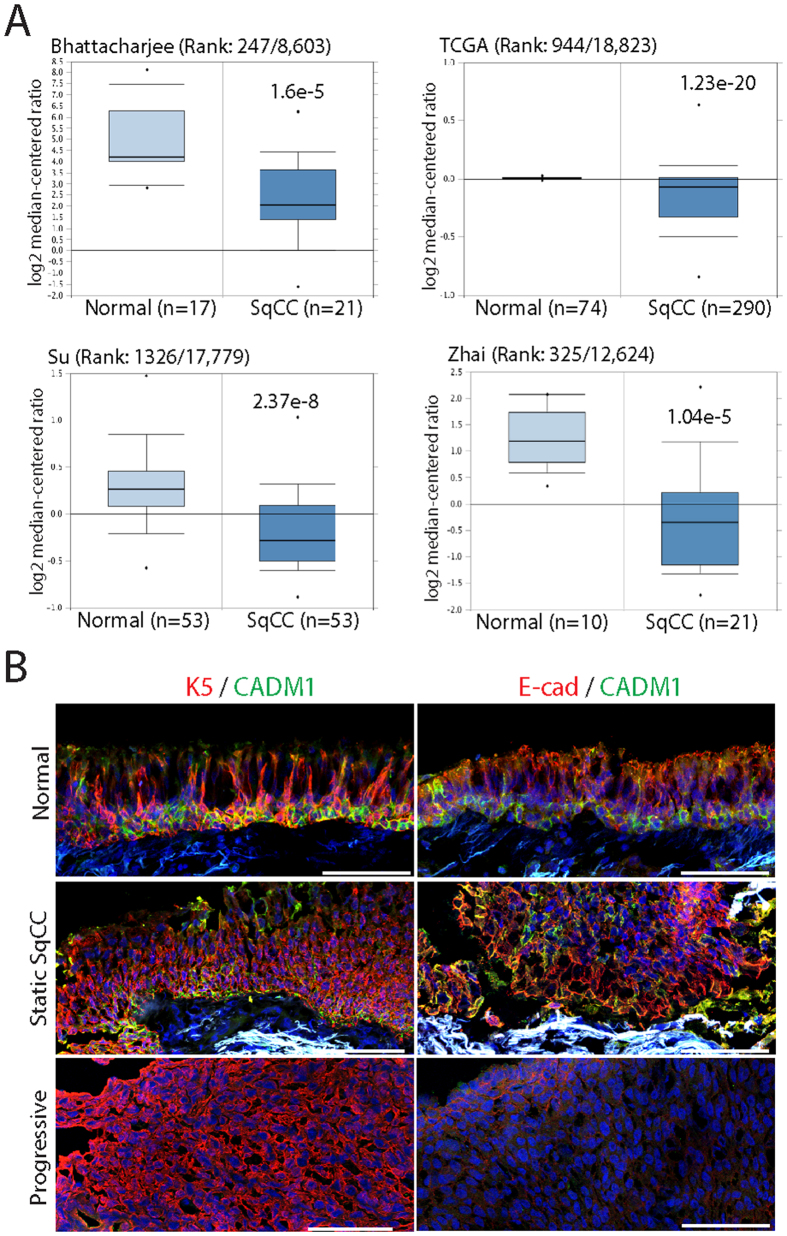
Loss of CADM1 is associated with squamous cell carcinoma progression. (**A**) Oncomine analysis of CADM1 expression in lung (Bhattacharjee), head and neck (TCGA), oesophagus (Su), and cervical (Zhai) SqCC datasets. Sample size (n), CADM1 gene rank, and p-values are shown for each tumour type. (**B**) Representative normal (n = 8), static (n = 9) and progressive (n = 10) lung SqCC biopsy specimens stained for keratin 5 (K5, red) plus CADM1 (green), or CADM1 (green) and E-cadherin (red). Scale bars are 100 μm (**B**) error bars denote standard error of the mean (**A**).

Previous studies have demonstrated that loss of CADM1 occurs in a majority of human SqCCs^8^,^9^,^10^. We verified these findings by analyzing CADM1 gene expression in normal and SqCC tissue samples representing lung, head and neck, oesophageal, and cervical cancers accessed through Oncomine (www.oncomine.org). Analysis of lung (Bhattacharjee), head and neck (TCGA), oesophagus (Su), and cervical SqCC (Zhai) datasets revealed significant reductions in CADM1 expression in all tumour samples relative to controls (Fig. 1A). A separate comparison of CADM1 gene expression in 3 normal keratinocyte (KJ, KV, KY) and 8 established SqCC cell lines (A431, SCC9, SCC12b, SCC13, SCC15, SCC27, SCC66, SCC71) revealed significant (>80%) or complete loss of CADM1 in 6 of 8 tumour cell lines compared with controls (Supplemental Figure 1).

To establish whether loss of CADM1 was specifically associated with SqCC progression we examined protein expression using a unique biobank of normal, static, and progressive SqCC samples obtained via autofluorescence bronchoscopy^18^,^19^. In some cases, patients’ lungs were under continuous surveillance for up to 10 years with no evidence of disease progression (static SqCC). In other cases patients’ tumours underwent rapid progression within 6–12 months (progressive SqCC). We identified epithelial tissues based on keratin 5 (Krt5) immunostaining (red, Fig. 1B) and assessed CADM1 and E-cadherin protein abundance in histologically normal lung (n = 8), static (n = 9), and progressive (n = 10) SqCC samples (green, Fig. 1B). In all normal lung and static SqCC biopsies, CADM1 and E-cadherin were readily detected throughout epithelial tissues (8/8 normal; 9/9 static SqCC samples; green, Fig. 1B). In contrast, CADM1 and E-cadherin staining were undetectable in the majority of progressive SqCC biopsies (8/10 lacking CADM1; 7/10 lacking E-cadherin; green, Fig. 1B). These results demonstrate that loss of CADM1 is associated with SqCC progression and coincides with the acquisition of EMT phenotypes including reduced E-cadherin expression.

### CADM1 inhibits SqCC cell growth and metastases

**Figure 2:**
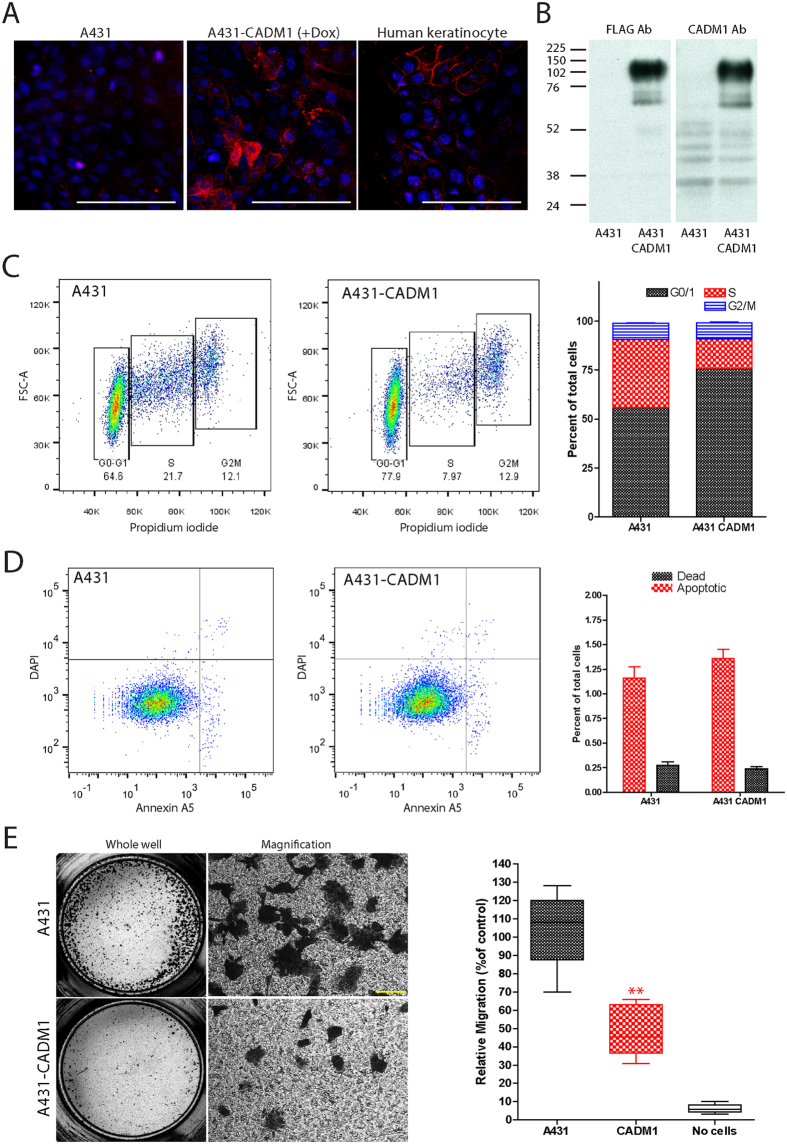
CADM1 expression reduces *in vitro* SqCC growth and migration. (**A,B**) Representative image (**A**) and protein immunoblot (**B**) of doxycycline treated wildtype and CADM1 transduced A431 cells. (**C**) Propidium iodide staining and quantification of cell cycle phase in control and CADM1 expressing cells. (**D**) DAPI and Annexin V staining and quantification of cell death and apoptosis in A431 control and CADM1 expressing SqCC cells. (**E**) Representative images of SqCC transwell migration and quantification of relative control and CADM1 expressing cell migration. Scale bars are 100 μm (**A,E**); asterisks denote significance of p < 0.005 (**); error bars denote standard error of the mean.

To determine whether CADM1 expression inhibits SqCC progression we studied its effects on the growth and migration of the CADM1-null SqCC cell line A431. Cells were transduced with a constitutively active luciferase reporter and a doxycycline-inducible CADM1-FLAG-IRES-GFP lentivirus cloned from a plasmid originally supplied by Prof. Y. Takai (Supplemental Figure 2). Doxycycline treatment of CADM1 transduced cells restored CADM1 gene and protein expression to physiologically relevant levels that were similar to those of noncancerous squamous epithelial cells (primary keratinocytes, Fig. 2A,B). In the absence of doxycycline, the growth and migration capacities of CADM1 transduced cells were indistinguishable from untransduced SqCC controls (data not shown). DNA cell cycle analysis of CADM1 transduced, doxycycline treated cells revealed that CADM1 restoration promoted accumulation of cells at the G0/G1 cell cycle checkpoint and reduced the number of S phase cells (Fig. 2C). In contrast, CADM1 expression had no effects on either SqCC cell death or apoptosis (Fig. 2D). Using transwell migration assays, we assessed the *in vitro* migration potential of CADM1 expressing tumour cells. Increased numbers of wildtype SqCC cells underwent transwell migration compared with doxycycline treated, CADM1-expressing cells (Fig. 2E).

**Figure 3:**
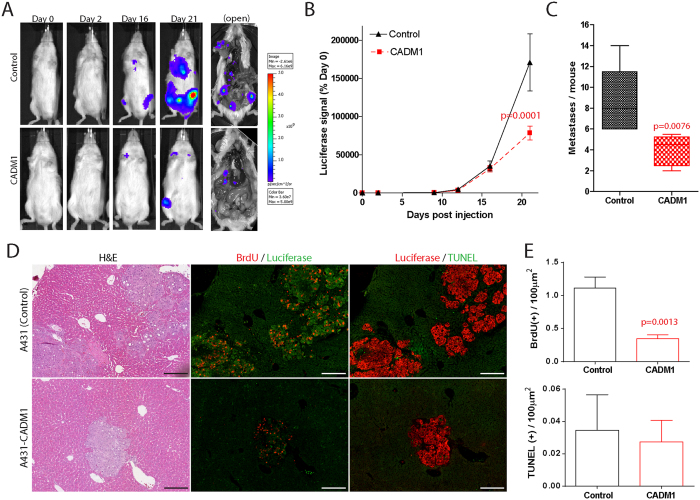
CADM1 inhibits SqCC progression. (**A**) Representative luciferase images of SqCC growth and metastatic burden in CADM1 negative (Control) and CADM1 expressing (CADM1) human tumour xenografts. (**B,C**) Quantification of tumour growth (% of luciferase signal relative to day 0, (**B**)) and metastatic burden (number of metastases/mouse, (**C**)) in control and CADM1 tumour xenografts (n = 8/cell type). (**D**) Sections of control and CADM1-expressing tumours stained with H&E, BrdU (red), luciferase (green), and TUNEL (green). (**E**) Quantification of BrdU(+) and TUNEL(+) cell abundance in control (black) and CADM1 expressing (red) tumours. Scale bars are 100 μm (**D**) error bars denote standard error of the mean.

To establish whether CADM1 inhibits SqCC progression *in vivo* we developed a human tumour xenograft model in which control or CADM1 expressing A431 cells were transplanted into immunocompromised mice by intravenous tail vein injection (n = 8 mice/tumour type). SqCC growth and metastases were monitored using noninvasive bioluminescent luciferase imaging. Immediately post injection, comparable luciferase signal strength was detected exclusively in the lungs of A431 control and A431-CADM1 tumour bearing mice (Fig. 3A,B). Longitudinal imaging demonstrated reduced SqCC growth in CADM1 expressing tumours relative to controls (p = 0.0001; Fig. 3A,B). Open imaging also revealed significantly fewer SqCC metastases in A431-CADM1 tumour bearing mice compared with untransduced SqCC controls (p = 0.0076; Fig. 3A,C). H&E and BrdU staining demonstrated reductions in A431-CADM1 tumour size and cell proliferation (Fig. 3D,E). As above, TUNEL staining revealed no differences in tumour cell apoptosis upon CADM1 reintroduction (Fig. 3D,E). Thus, CADM1 inhibits SqCC progression both *in vitro* and *in vivo* by reducing cell proliferation and invasion.

### The extracellular domain of CADM1 regulates SqCC progression

**Figure 4:**
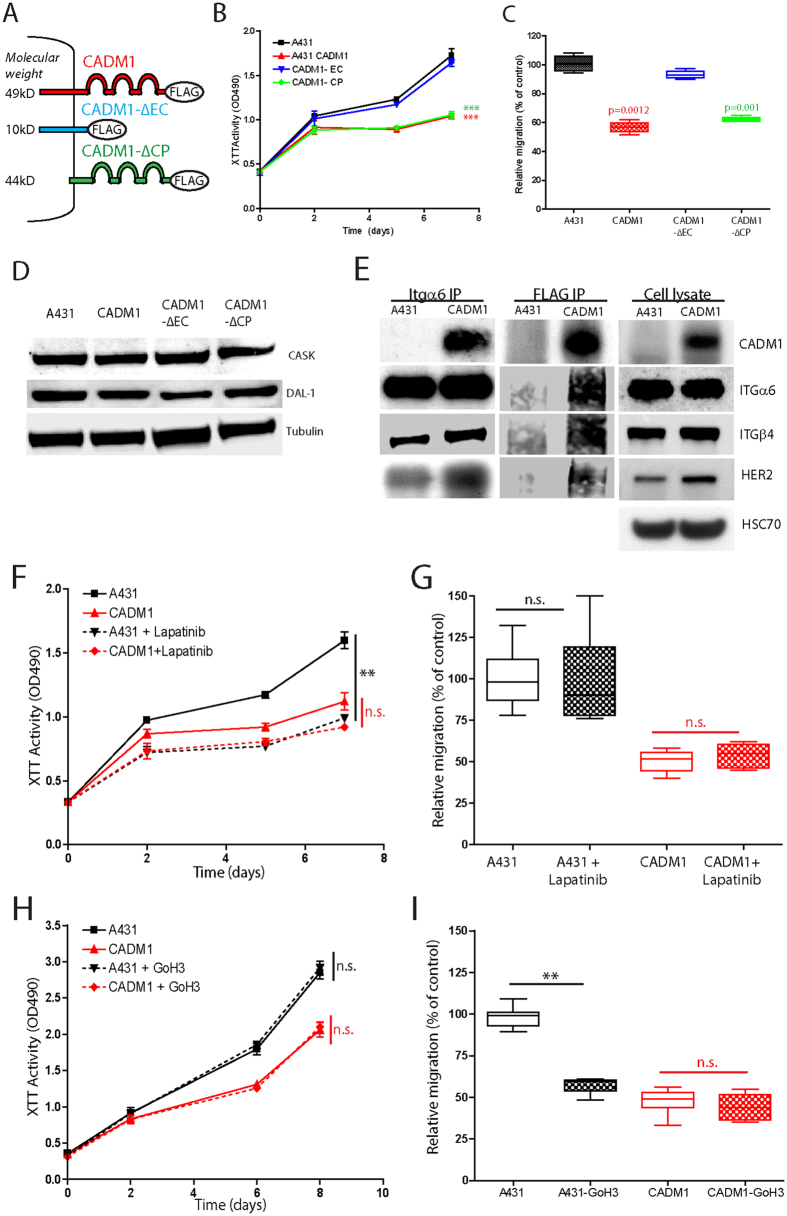
The extracellular domain of CADM1 determines SqCC progression. (**A**) Schematic of full length, extracellular (CADM1-ΔEC) and cytoplasmic (CADM1-ΔCP) domain deletion constructs. (**B,C**) *In vitro* control, CADM1, CADM1-∆EC and CADM1-∆CP expressing cell proliferation (**B**) and migration (**C**). (**D**) Immunoblot of CASK and Dal-1 abundance in A431 control, Cadm1, ΔEC, and ΔCP expressing cell lines. (**E**) Immunoprecipitation (IP) of control and CADM1-expressing SqCC lysates using anti-FLAG and anti-ITGα6 antibodies. IP and total cell lysates were probed with antibodies as indicated. (**F–I**) Graphs of control (A431) and CADM1-expressing SqCC proliferation (**F,H**) and cell migration (**G,I**) following treatment with Lapatinib (**F,G**) or GoH3 (**H,I**). n = 3 per cell type and assay (**B,C,F–I**); asterisks denote significance of p < 0.005 (**) or p < 0.001 (***); error bars denote standard error of the mean. IP experiments (**E**) were performed twice using independent cell lysates.

Conflicting evidence surrounds the relative importance of the extracellular (EC) versus cytoplasmic (CP) signaling domains of CADM1 in regulating tumour progression^11^,^14^,^15^. To identify whether EC or CP domains of CADM1 regulate SqCC growth and metastases, we transduced A431 cells with CADM1 mutant constructs lacking either their extracellular (∆EC) or cytoplasmic (∆CP) signaling domains (originally described in^14^,^15^, Fig. 4A). CADM1-ΔEC expression resulted in SqCC growth and migration that was indistinguishable from untransduced CADM1-null controls (compare blue and black lines and bars, Fig. 4B,C). In contrast, the growth and migration of CADM1-ΔCP expressing cells was equivalent to that of full length CADM1 expressing cells (compare red and green bars and lines; Fig. 4B,C). As above, we used a human tumour xenograft model to assess CADM1-ΔCP and CADM1-ΔEC growth and metastases *in vivo*. Consistent with our *in vitro* results, longitudinal and open imaging demonstrated that CADM1-ΔCP tumours grew at a reduced rate and with fewer metastases per mouse when compared with CADM1-ΔEC tumours (*p < 0.01; n = 6 animals/cell type; Supplemental Figure 3).

To validate these results, we analyzed the expression of key CADM1 cytoplasmic signaling proteins CASK and DAL-1 in control, CADM1, ΔCP and ΔEC cell lines. As expected, there were no significant differences in CASK or DAL-1 expression between any of these cell types (Fig. 4D). These results confirm that the extracellular portion of CADM1 regulates SqCC progression independent of cytoplasmic CASK and DAL-1 signaling.

Earlier studies have demonstrated that the extracellular domain of CADM1 can interact with both integrin α6β4 and HER2^14^,^15^. To identify whether one or more of these interactions might regulate SqCC progression, we used integrin α6 and CADM1 immunoprecipitation (IP) assays. In SqCC controls, Itgα6, integrin β4 (Itgβ4), and HER2 formed a HER2-Itgα6-Itgβ4 protein complex independent of CADM1 expression (Fig. 4E; uncropped cell lysate blots in Supplemental Figure 4). In contrast, immunoprecipitation of CADM1 expressing cells using an anti-FLAG antibody demonstrated the presence of an additional complex involving CADM1, Itgα6, Itgβ4, and HER2 (Fig. 4E). All IP experiments were performed twice using independently generated cell lysates.

To determine which of the specific interactions between CADM1, HER2, and Itgα6β4 influenced SqCC growth and metastases, cells were treated with Lapatinib, a small molecule inhibitor of HER2, and GoH3, a monoclonal antibody that blocks the functional domain of integrin α6. Lapatinib treatment reduced the growth of untransduced SqCC cells to a level identical to that of CADM1 expressing cells but had no effect on cell migration (black, Fig. 4F,G). In contrast, GoH3 had no effect on control SqCC cell growth but did reduce cell migration (black, Fig. 4H,I). Neither Lapatinib nor GoH3 had any effects on SqCC cell growth or migration when CADM1 was expressed, confirming the specificity of these effects to CADM1-null cells (red, Fig. 4F–I). These results suggest that CADM1 inhibits SqCC growth and motility through a mechanism involving both HER2 and Itgα6β4 downstream signaling.

### CADM1 inhibits SqCC progression by reducing STAT3 activity

**Figure 5:**
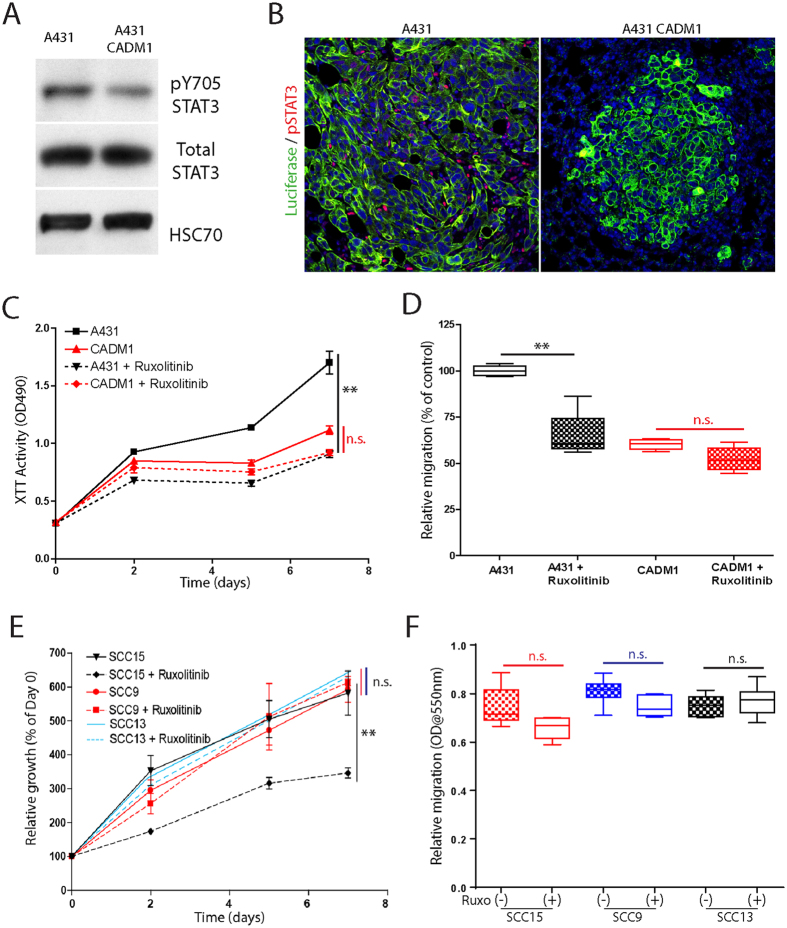
CADM1 reduces SqCC STAT3 activity. (**A,B**) Immunoblot (**A**) and immunofluorescent staining ((**B**), red) of active STAT3 (pY705) abundance in control and CADM1-expressing SqCC. (**C,D**) Graphs of control (A431) and CADM1-expressing SqCC proliferation (**C**) and cell migration (**D**) following treatment with 10 μm ruxolitinib. (**E,F**) Proliferation (**E**) and migration (**F**) of three additional, endogenous CADM1-expressing SqCC cell lines (SCC9, red; SCC13, blue; SCC15, black) in the absence (solid lines) or presence (dashed lines, hollow boxes) of ruxolitinib treatment. Error bars represent SEM (n = 4/condition); asterisks denote significance of p < 0.005 (**). STAT3 immunoblots are representative of n = 3 independent experiments.

It has previously been shown that Itgα6β4 can cooperate with HER2 to drive downstream STAT3 phosphorylation and signaling^20^,^21^. We therefore examined whether CADM1 interactions with HER2 and Itgα6β4 in SqCC might regulate STAT3 phosphorylation. Untransduced and CADM1-expressing A431 cells were grown to 70% confluence, serum starved overnight, and treated with EGF for 15 minutes to induce STAT3 activation prior to immunoblotting. Phospho-specific tyrosine 705 (pY705) STAT3 staining and ImageJ quantification demonstrated that CADM1 expression reduced A431 STAT3 phosphorylation levels to 41 ± 6% of untransduced controls (n = 3, Fig. 5A). We also observed a complete lack of phospho-STAT3 staining in CADM1-expressing SqCC tumour xenografts (red, Fig. 5B). This difference between *in vitro* and *ex vivo* A431-CADM1 STAT3 activity may relate to fundamental differences in these two model systems.

To examine whether CADM1 expression might inhibit SqCC progression via direct STAT3 inhibition, CADM1 null, CADM1 transduced, and endogenous CADM1 expressing SqCC cells were treated with the clinically approved JAK/STAT inhibitor, ruxolitinib. In CADM1 null A431 cells, ruxolitinib treatment reduced both *in vitro* cell growth and migration (black, Fig. 5C,D). In contrast, ruxolitinib treatment had no effects on CADM1 transduced A431 cells, verifying the specificity of these effects in SqCC cells lacking CADM1 (red, Fig. 5C,D). In a SqCC cell line with low endogenous CADM1 expression (SCC15, Supplemental Figure 1), ruxolitinib treatment significantly reduced cell growth but had no effect on cell migration (black, Fig. 5E,F). In two further SqCC cell lines with robust endogenous CADM1 expression (SCC9 and SCC13, Supplemental Figure 1), ruxolitinib treatment had no effect on either SqCC cell growth or migration (red and blue lines, Fig. 5E,F). These results suggest that endogenous CADM1 expression determines tumour ruxolitinib sensitivity, although other pathways may also be involved.

**Figure 6:**
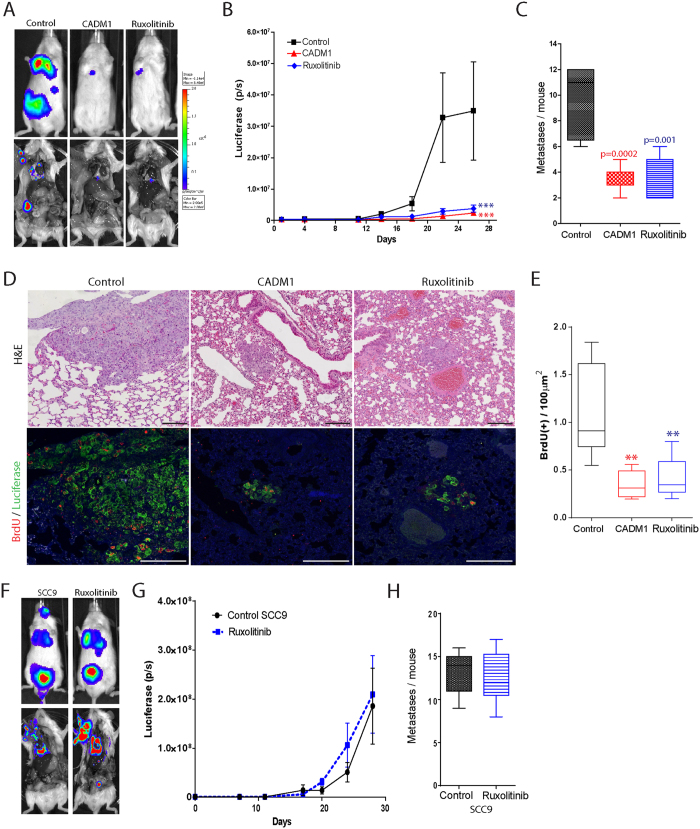
CADM1-null SqCC are sensitive to JAK/STAT chemotherapy. (**A**) Representative images of SqCC growth and metastatic burden in human tumour xenografts following treatment with ruxolitinib or restoration of CADM1 expression (n = 6/treatment type). (**B,C**) Quantification of SqCC growth (**B**) and metastatic burden (**C**) in control (black), CADM1 expressing (red), and ruxolitinib treated tumours (blue). (**D**) Control, CADM1-restored, and ruxolitinib treated tumour sections stained with H&E, BrdU (red), and luciferase antibodies (green). (**E**) Quantification of BrdU(+)cell abundance in control (black), CADM1 expressing (red), and ruxolitinib treated tumours (blue). (**F**) SCC9 tumour size and metastatic burden in human tumour xenografts with or without ruxolitinib treatment. (**G,H**) Quantification of SCC9 tumour growth (**G**) and metastatic burden (**H**) in the absence (black, n = 7) or presence of ruxolitinib (blue, n = 7). Scale bars are 100 μm (**D**) asterisks denote significance of p < 0.005 (**) or p < 0.001 (***); error bars denote standard error of the mean.

Based on our *in vitro* results, we investigated whether *in vivo* treatment with the JAK/STAT inhibitor ruxolitinib could mimic the effects of restoring CADM1 expression on reducing CADM1-null SqCC progression. As before, we used a tumour xenograft model of luciferase transduced, CADM1 negative and CADM1 expressing A431 SqCC cells. Longitudinal tumour imaging revealed that ruxolitinib significantly and specifically reduced CADM1-null SqCC growth and metastases to a level that was indistinguishable from CADM1 expressing cells (Fig. 6A–C). Further H&E and BrdU staining analysis demonstrated that treatment with ruxolitinib achieved this effect by reducing SqCC proliferation (Fig. 6D,E).

To establish whether endogenous CADM1 expression could influence *in vivo* SqCC sensitivity to JAK/STAT inhibition we generated human tumour xenografts using the SqCC lines SCC9 and SCC13. Consistent with our *in vitro* results, ruxolitinib treatment had no effect on either the growth or metastatic potential of SCC9 and SCC13 tumour xenografts (Fig. 6F–H and data not shown). These results demonstrate that treatment with the JAK/STAT inhibitor ruxolitinib might only be an effective therapy in SqCC tumours that lack endogenous CADM1 expression.

## Discussion

**Figure 7:**
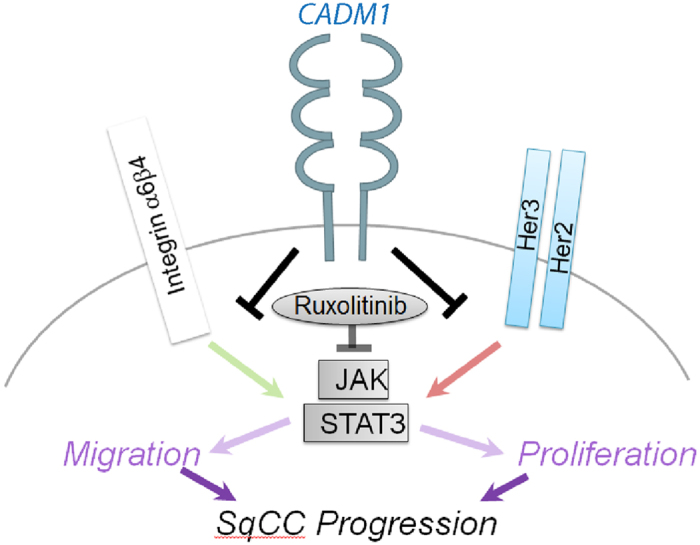
Model of CADM1 activity in SqCC. In CADM1-null tumours HER2 and ITGα6β4 cooperate to drive STAT3-mediated tumour growth and metastases. CADM1 promotes a CADM1-HER2-ITGα6β4 ternary complex that suppresses downstream STAT3 activity thereby inhibiting cell proliferation and migration. Ruxolitinib mimics the effects of CADM1 in suppressing HER2-ITGα6β4 mediated STAT3 activity.

Despite recent advances in murine models for human SqCC, an understanding of the mechanisms that drive tumour progression remains elusive. Here, we demonstrate that CADM1 inhibits SqCC progression via a mechanism involving HER2 and ITGα6β4-mediated STAT3 inhibition (Fig. 7).Our results indicate that the extracellular domain of CADM1 is responsible for suppressing tumour progression, consistent with previous studies of human colorectal cancer cells^14^,^15^. Using tumour xenograft studies, we demonstrate that the extracellular domain of CADM1 regulates SqCC progression via generation of a HER2-ITGα6β4-CADM1 complex at the cell surface that inhibits HER2-ITGα6β4 mediated STAT3 phosphorylation. We also establish that treating CADM1 negative cancer cells with the clinically approved JAK/STAT inhibitor ruxolitinib can reduce *in vitro* and *ex vivo* tumour growth and metastases. These results suggest that screening human SqCCs for CADM1 expression may not only help identify patients at risk of disease progression but also distinguish those people most likely to benefit from JAK/STAT targeted chemotherapies.

There is a significant, growing interest in the use of JAK/STAT and STAT3-specific therapies to treat human SqCC. In preclinical and early phase clinical studies, STAT3 oligonucleotide decoys as well as JAK small molecule inhibitors have shown promise in head and neck, lung, and other SqCC types^22^,^23^,^24^. Despite this, separate studies suggest that STAT3 may also play a tumour suppressive role in some cancers^25^,^26^ and that STAT3 deletion can in fact promote lung adenocarcinoma formation^27^. In light of this, ours is the first study to demonstrate that loss of endogenous CADM1 expression may serve as a novel biomarker for determining JAK/STAT chemotherapy responsiveness.

Our results may explain why single chemotherapy agents that target receptor tyrosine kinases such as HER2 have largely proven ineffective in treating SqCC^28^. Specifically, our discovery that inhibiting either HER2 or ITGα6β4 alone is ineffective at blocking *in vitro* SqCC growth and migration suggests that targeting either molecule on its own may inadvertently promote downstream STAT3 activity. In support of this possibility, previous studies have shown that integrin α6β4 cooperates with HER2 to drive STAT3 activation during tumour progression^20^. It has also recently been shown that single pharmacological inhibitors of EGFR, MEK, PI3K and FGFR increase both STAT3 activity and tumour metastases in various cancer models^29^,^30^,^31^.

Although our study establishes that CADM1 inhibits SqCC progression, the mechanisms driving the initial loss of CADM1 expression remain incompletely understood. In many human cancers, CADM1 silencing occurs following promoter methylation and loss of chromosomal heterozygosity^8^,^9^,^10^. Increased transcription of the miRNA cluster encoding miR-214 and miR-199 has also been shown to reduce CADM1 expression via loss of ST6GAL1-dependent CADM1 sialylation^32^. Interestingly, transcription of this miRNA cluster is itself regulated by TWIST1, an EMT biomarker associated with SqCC progression and canonical Wnt/β-catenin target gene activation^33^,^34^. Given that we have recently shown that SqCC pathogenesis is associated with increased Wnt/β-catenin signaling^16^, we believe a process involving increased Wnt/β-catenin, acquisition of EMT phenotypes, and reduced ST6GAL1-dependent sialylation is a plausible mechanism to drive epigenetic loss of CADM1 in human SqCC.

In previous studies, CADM1 has been shown to promote immune surveillance via a mechanism involving Class 1 restricted, T cell associated molecule (CRTAM) engagement^12^,^13^,^35^. In the current study, our use of immunocompromised mouse models excludes a role for CADM1-CRTAM interactions in regulating SqCC progression. However, we speculate that in human patients CRTAM-dependent immune surveillance of CADM1 expressing tumours may provide an additional mechanism by which CADM1 suppresses tumour growth. Interestingly, immune surveillance is emerging as a key factor in SqCC ontogeny^28^,^36^, with increased expression of PD-1, PD-L1, and CTLA-4 reducing immune surveillance in multiple tumour types^12^,^13^,^36^,^37^,^38^. Consistent with this, clinical trials involving PD-1, PD-L1 and CTLA4 monoclonal antibody therapies have recently shown promise in patients with SqCC^28^,^37^. In light of these findings, our data suggests that combinatorial therapies involving increased immune surveillance (perhaps via CTLA-4, PD-1, or PD-L1 inhibition) coupled with targeted JAK/STAT chemotherapies may prove beneficial in patients with CADM1 negative disease.

## Additional Information

**How to cite this article**: Vallath, S. *et al.* CADM1 inhibits squamous cell carcinoma progression by reducing STAT3 activity. *Sci. Rep.* **6**, 24006; doi: 10.1038/srep24006 (2016).

## Supplementary Material

Supplementary Information
