# Supplementary material for: CADM1 inhibits squamous cell carcinoma progression by reducing STAT3 activity
Source: Sci Rep. 2016 Apr 1;6:24006. doi: 10.1038/srep24006 (PMC4817512; doi:10.1038/srep24006)
Supplement: Supplementary Information [file srep24006-s1.pdf]

Supplemental information:

CADM1 inhibits squamous cell carcinoma progression by reducing STAT3 activity

Sabari Vallath (1), Elizabeth K Sage (1), Krishna K Kolluri (1), Sofia N Lourenco (1), Vitor S Teixeira (1), Suneeta Chimalapati (1), P Jeremy George (1), Sam M Janes (1), Adam Giangreco (1,2)

Supplemental Figure 1.

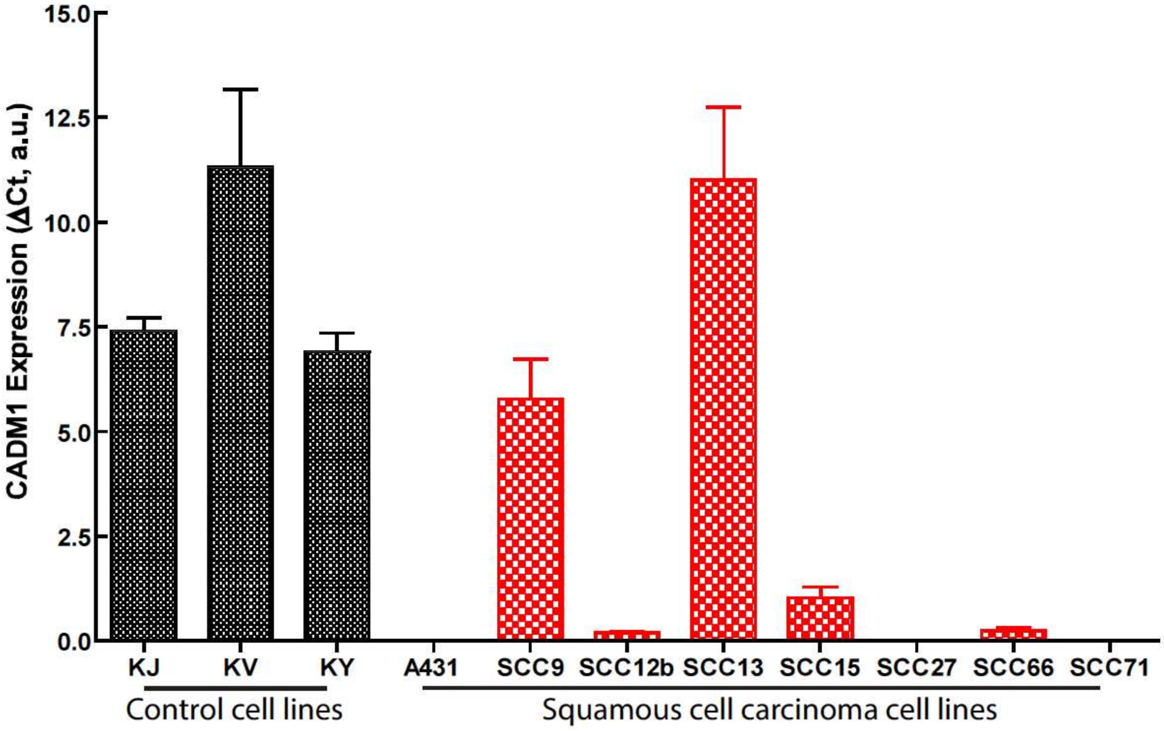

Loss of Cadm1 is common in human SqCC cell lines. CADM1 RNA expression abundance in normal (n=3) and SqCC cell lines (n=8).

Supplemental Figure 2.

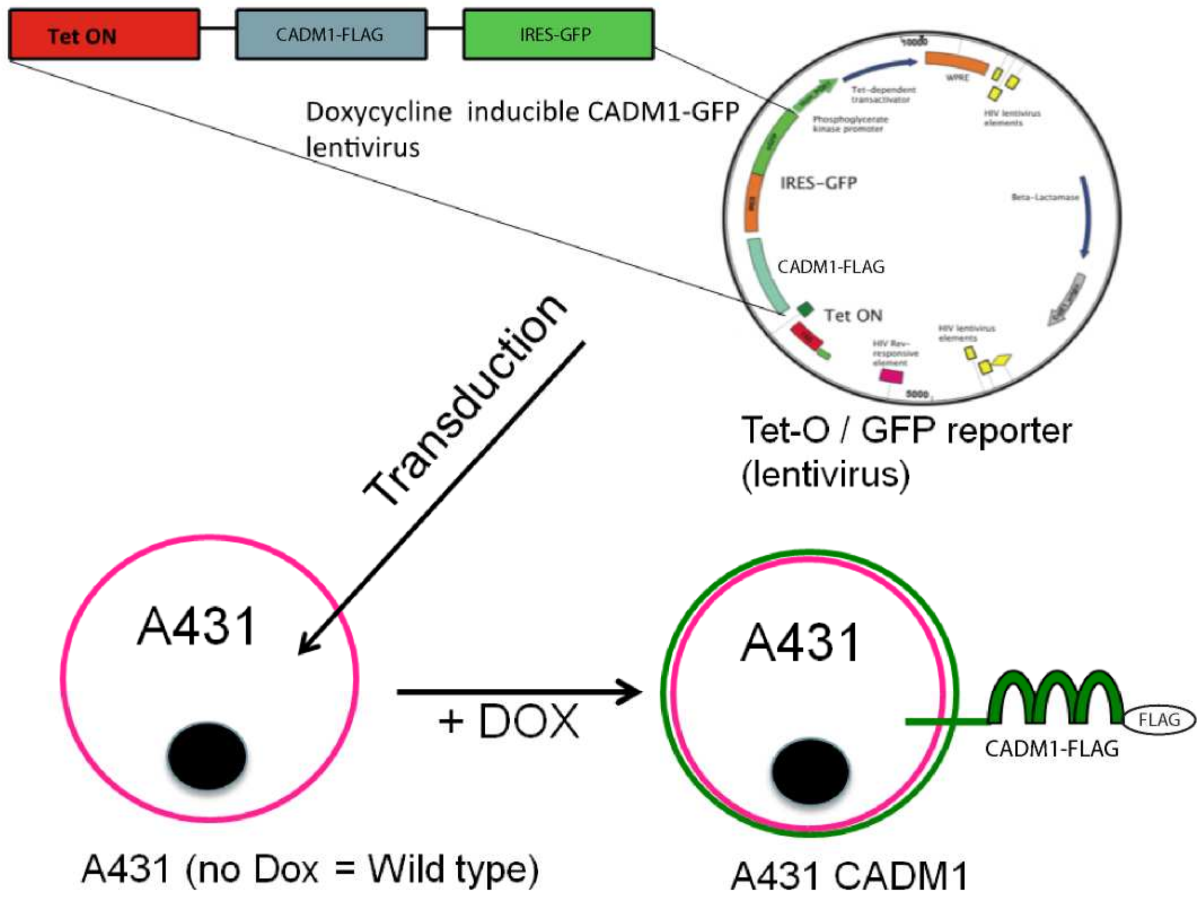

Schematic map of doxycycline-inducible CADM1-IRES-GFP lentivirus. Doxycycline treatment of CADM1 transduced SqCC cells (+DOX in schematic) restores CADM1 gene and protein expression to physiologically relevant levels.

Supplemental Figure 3.

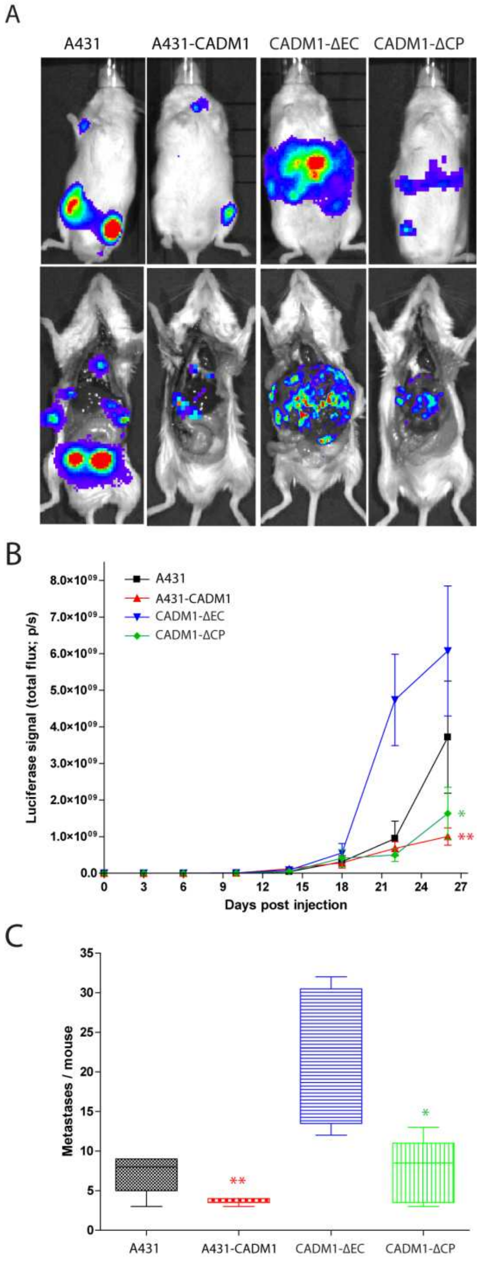

(A) Representative images of SqCC growth and metastatic burden in control, CADM1, CADM1-ΔEC, and CADM1-ΔCP SqCC xenografts (n=4 / human tumour xenograft type). (B, C) Quantification of SqCC growth (luciferase signal intensity, B) and metastatic burden (C) for all tumour types. Error bars (B, C) represent standard error of the mean; asterisks denote significance of $p<0.005$ (**) or $p<0.05$ (*).

Supplemental Figure 4.

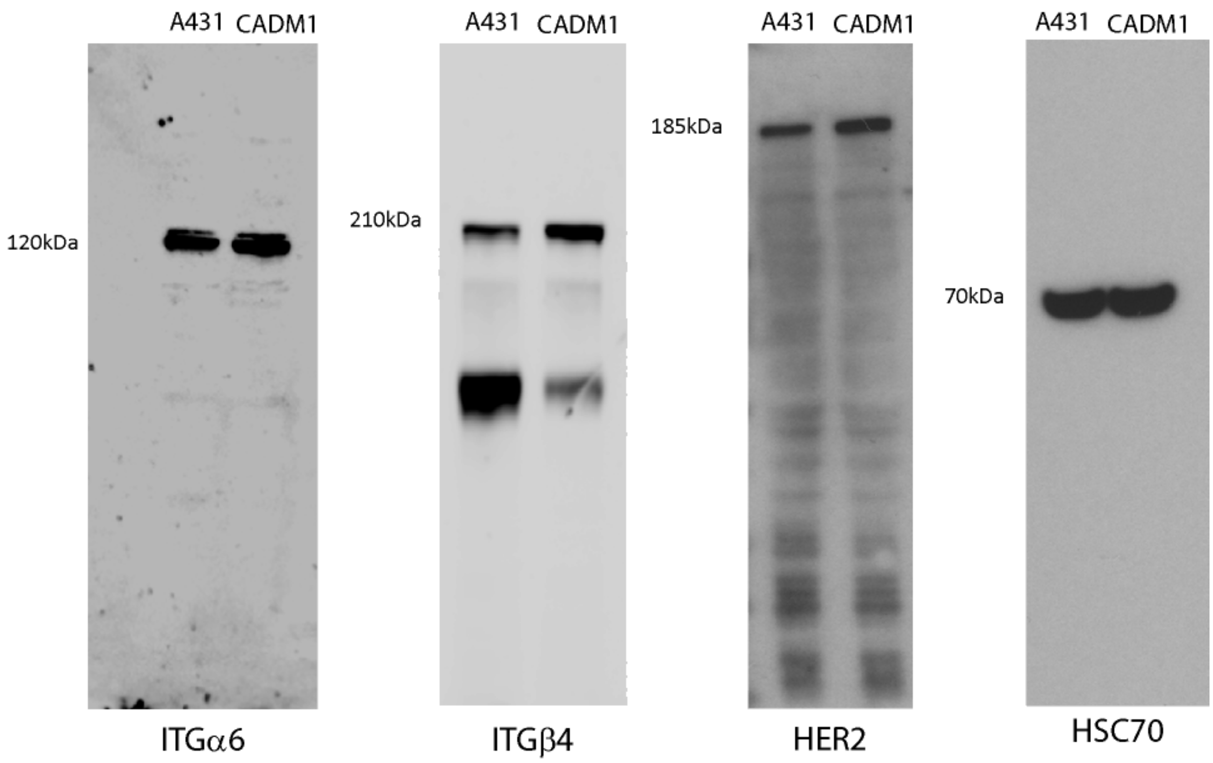

Uncropped western blots of cell lysates used in Figure 4E. The molecular weight of each band corresponding to the predicted protein size is indicated.
